# Peptide receptor radionuclide therapy in advanced Pheochromocytomas and Paragangliomas: a systematic review and meta-analysis

**DOI:** 10.3389/fonc.2023.1141648

**Published:** 2023-07-06

**Authors:** Dan Su, Hongyu Yang, Chen Qiu, Yue Chen

**Affiliations:** ^1^ Department of Nuclear Medicine, The Affiliated Hospital of Southwest Medical University, Luzhou, Sichuan, China; ^2^ Nuclear Medicine and Molecular Imaging Key Laboratory of Sichuan Province, Luzhou, Sichuan, China; ^3^ Department of Ophthalmology, The Affiliated Hospital of Southwest Medical University, Luzhou, Sichuan, China; ^4^ Academician (expert) Workstation of Sichuan Province, Luzhou, Sichuan, China

**Keywords:** pheochromocytomas, paragangliomas, PPGLs, PRRT, meta-analysis

## Abstract

**Objective:**

Peptide receptor radionuclide therapy (PRRT) for advanced pheochromocytomas and paragangliomas (PPGLs) has received increasing attention. The purpose of this article is to evaluate the efficacy and safety of PRRT in patients with metastatic or inoperable PPGLs by meta-analysis.

**Methods:**

A literature search was conducted in PubMed, Embase, Scopus, and Cochrane Library databases up to November 2022. All articles on PRRT for PPGLs were searched, and appropriate data were included for analysis. The measures evaluated included objective response rate (ORR), disease control rate (DCR), clinical response rate, biochemical response rate, progression-free survival (PFS), overall survival (OS), and adverse events. Statistical analysis was performed using Stata 16.0 and the R programming language, data were combined using a random-effects model, and the results were presented using forest plots.

**Results:**

A total of 20 studies with 330 patients were included in the analysis. The results showed that ORR and DCR were 20.0% (95% CI: 12.0%-28.0%) and 90.0% (95% CI: 85.0%-95.0%), respectively. Clinical and biochemical responses were 74.9% (95% CI: 56.3%-90.2%) and 69.5% (95%CI: 40.2%-92.9%). Median PFS and median OS were 31.79 (95% CI:21.25-42.33) months and 74.30 (95% CI: 0.75-147.84) months, respectively. Any grade of hematotoxicity and nephrotoxicity occurred in 22.3% (95% CI:12.5%-33.5%) and 4.3% (95% CI:0.2%-11.4%) patients. Grade 3-4 hemotoxicity occurred in 4.3% (95% CI:0.2%-11.4%) and grade 3-4 nephrotoxicity in 4/212 patients. Additionally, Treatment was discontinued in 9.0% (95% CI: 0.5%-23.3%) patients and one patient died as a result of a toxicity.

**Conclusion:**

Patients with metastatic or inoperable PPGLs can be effectively treated with PRRT, and it has a favorable safety profile.

**Systematic review registration:**

https://www.crd.york.ac.uk/PROSPERO, identifier CRD42022359232.

## 1 Introduction

Pheochromocytomas (PCCs) originate from the adrenal medulla and paragangliomas (PGLs) originate from the paraganglia of the sympathetic or parasympathetic nerves located outside the adrenal glands, both of which are rare types of neuroendocrine tumors (1, 2). Metastatic pheochromocytomas and paragangliomas (MPPGLs) are defined as tumors found outside the adrenal medulla or paraganglion tissue (3). Approximately 10% of PCCs and 34% of PGLs present with metastases at initial diagnosis, which can occur many years after initial diagnosis (4, 5). PPGLs usually metastasize to lymph nodes, bones, the liver, and the lungs (6–8). The median 5-year overall survival of metastatic disease is only about 60%, and its prognosis is usually dismal (8). Treatment of MPPGLs is challenging, with most MPPGLs progressing slowly and patients with progressive MPPGLs requiring systemic therapy (8, 9). Systemic chemotherapy and ^131^I-MIBG therapy often have significant hematotoxicity (10, 11). Recent approval of ^177^Lu-DOTATATE by the US Food and Drug Administration (FDA) for the treatment of well-differentiated inoperable/metastatic gastrointestinal-pancreatic neuroendocrine tumors (GEP-NETs) and clinical trial of metastatic or inoperable PPGLs has increased awareness of Peptide receptor radionuclide therapy (PRRT) for the treatment of PPGLs expressing somatostatin analog receptor (SSTR). SSTR imaging is typically used to select PRRT candidates (12). The most commonly used radionuclides for PRRT are ^177^Lu and ^90^Y, which release β rays to cause cell damage and achieve the therapeutic effect. However, a novel therapy option is the use of radionuclides with high linear energy transfer (LET) α emission, like ^225^Ac and ^213^Bi, as there are still a lot of patients who do not respond to the available choices. Since ^225^Ac has a much longer half-life (240 hours) and emits a lot of particles, it is much more cytotoxic than the emitter ^213^Bi, which has a very short half-life (46 minutes) (13). Patients with NETs who do not respond to ^177^Lu-DOTATATE or who have completed the recommended number of treatment cycles have been demonstrated to have better outcomes with ^225^Ac-DOTATATE (14). There have been recent developments in the usage of PRRT for advanced PPGLs, particularly the use of ^225^Ac in PRRT to offer new choices (15, 16). We, therefore, conducted this meta-analysis to assess the efficacy and safety of PRRT in patients with advanced (metastatic or inoperable) PPGLs.

## 2 Materials and methods

The systematic review followed the Preferred Reporting Items for Systematic Reviews and Meta-Analysis (PRISMA) guidelines (17).

The registration number on the International Prospective Register of systematic reviews (PROSPERO) is: CRD42022359232.

### 2.1 Literature search strategy

Two authors (DS and HY) independently conducted a systematic literature search in PubMed, Embase, Scopus, and Cochrane Library databases. The time frame of the systematic literature search is from the establishment to November 2022. Search terms included, “Pheochromocytoma”, “Pheochromocytomas”, “Paraganglioma”, “Paragangliomas”, “peptide receptor radionuclide therapy”,”PRRT”,”^177^Lu”,”Lu-177”, “Lutetium-177”, “^177^Lutetium”,”^90^Y”,”Y-90”,”Yttrium-90”,”^90^Yttrium”,”^225^Ac”,”Ac-225”,”Actinium-225”,”^225^Actinium”. All original articles were searched and relevant data were included for analysis. The full text was searched if the article met the study criteria.

### 2.2 Study selection

We only selected studies that meet the following criteria: Participants (P) were no less than 5 people who had been diagnosed as inoperable or metastatic pheochromocytoma or paraganglioma. Interventions (I) were completed at least one cycle of PRRT with no restrictions on prior treatment; If data came from the same study group, the study with the highest number of patients will be included. The main outcome endpoint (O) was objective response rate (ORR) and disease control rate (DCR) are assessed by response evaluation criteria in solid tumors (RECIST) or Southwest Oncology Group (SWOG) criteria (18, 19). The type of study (S) included in the article was retrospective or prospective research. Exclusion criteria include: non-clinical studies; the number of patients <5; multiple treatments are carried out simultaneously; reviews, meta-analysis, case reports, letters to editors, conference abstracts, articles on biodistribution and dosimetry, articles reporting only adverse events; and articles for which the English text was not available.

### 2.3 Quality evaluation

Two authors (DS and HY) separately evaluated the quality of each article included in the analysis, and any disagreements were resolved by consensus. The quality of cohort studies was assessed by the Newcastle-Ottawa Quality Assessment Scale used for cohort studies. The Newcastle-Ottawa Quality Assessment Scale contains three sections with a total of nine assessment elements, each of which can be rated as one star if the study is of high quality for the particular element, for a total score of 9 stars. High-quality studies were those that obtained 6 or more stars (20).

### 2.4 Data extraction

Two authors (DS and HY) independently reviewed all articles eventually included in the analysis and extracted pertinent data, and any disagreements were resolved by consensus. First author, year of publication, study type, patient demographics, PRRT treatment parameters, including radiopharmaceuticals, radiopharmaceutical activity, number of treatment cycles, duration of follow-up, and adverse events were all included in the basic data that was extracted for all studies. The ORR and DCR were the primary outcome endpoints. Clinical response rate, biochemical response rate, progression-free survival (PFS), overall survival (OS), and adverse events were secondary outcome endpoints.

ORR was defined as the percentage of complete response (CR) and partial response (PR). DCR was the proportion of CR, PR and stable disease (SD). Clinical response rate was the percentage of any improvement in tumor-related symptoms and/or decrease in relevant drug dosage after treatment. The biochemical response rate was the proportion of patients with any decline in tumor markers. PFS was the period, measured in months, between the first PRRT and the commencement of progression or death. OS was the period, measured in months, between the first PRRT and death from any cause. The median, mean, and 95% confidence intervals for PFS and OS were extracted from individual articles. Toxicity was defined according to the Common Terminology Criteria for Adverse Events Version 5.0 (CTCAE V5.0) (21).

### 2.5 Statistical analysis

Stata16.0 and the R programming language was used for meta-analysis. Because of the clinical heterogeneity in study designs, random-effects models were employed for the computations, and the findings of the meta-analysis were displayed as forest plots. Statistical heterogeneity between studies was assessed using the Cochran Q test and Higgins I^2^ statistics. Heterogeneity is deemed significant if the P < 0.1 or I^2^ ≥ 50%. Heterogeneity is regarded as insignificant, if the P ≥ 0.1 and the I^2^ < 50%.

## 3 Results

### 3.1 Literature search and screening

**Figure 1:**
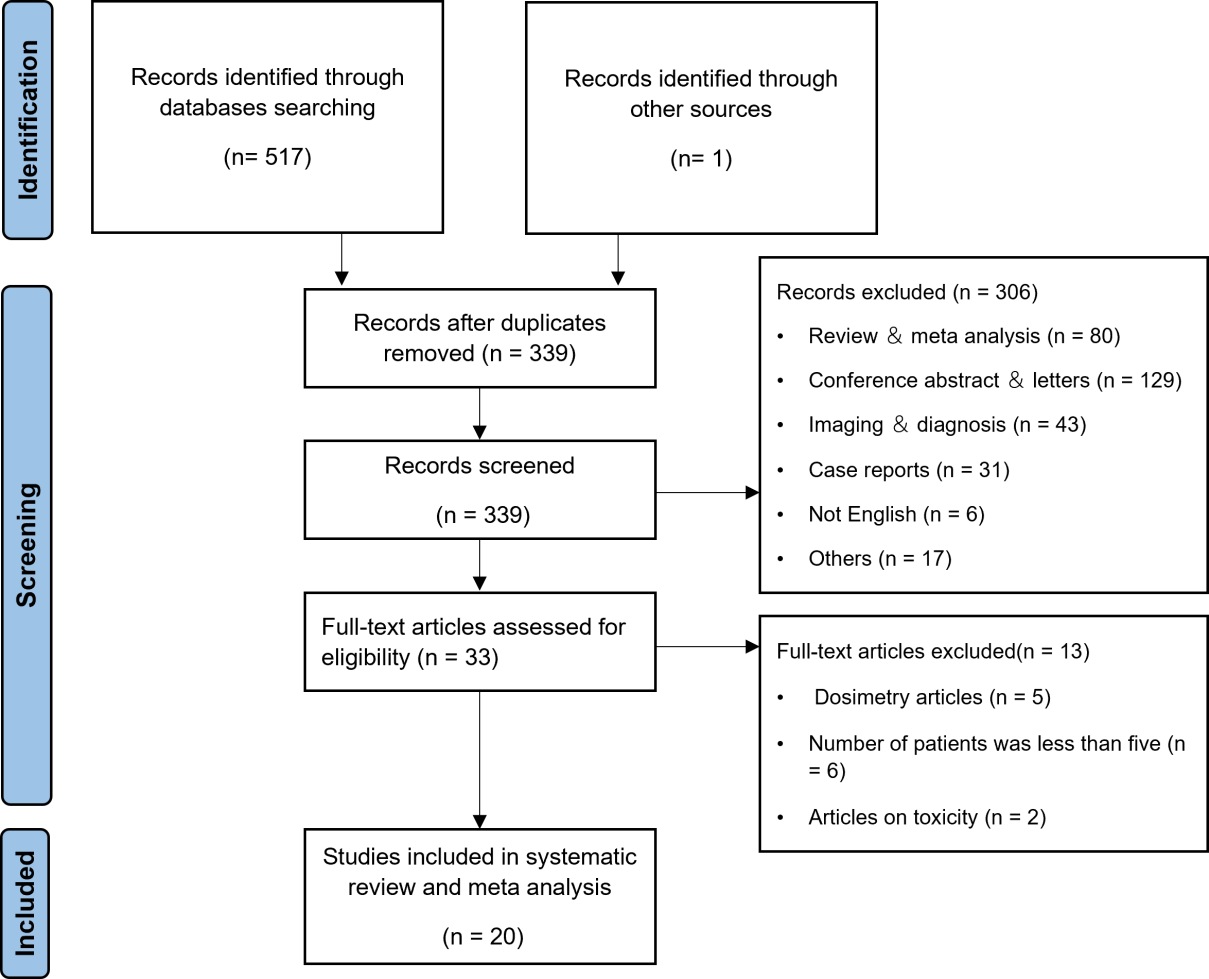
Study selection process.

According to the search strategy, a total of 517 articles were retrieved, and 338 articles remained after excluding duplicate articles. 306 articles were excluded by title and abstract. After reading through the full text, 13 articles were excluded by the inclusion and exclusion criteria. One more article was found for inclusion throughout the full-text review process by manually searching all references. In the end, 20 articles (16, 22–40) were included in the meta-analysis, as shown in **Figure 1**.

### 3.2 Characteristics and quality of the studies

A total of 330 patients were included in the 20 articles that made up the meta-analysis published between 2006 and 2022. The detailed characteristics of the included studies are shown in **Table 1**. The included studies were all cohort studies and were assessed for quality using the Newcastle-Ottawa Quality Assessment Scale. All the included studies were of good quality. The evaluation of the included studies’ quality is shown in **Table 2**.

**Table 1:** Characteristics of included studies.

First author	Year	Study type	No. of Patients	Age (years) (Median, Range)	Sex	PRRT agent	Activity (GBq/cycle)	Cycles of Therapy (Median, Range)	Follow-up (Month) (Median, Range)
Yadav (16)	2022	R	9	41 (23–65)	M 6, F 3	^225^Ac	100kBq/kg	3 (2-9)	22.5
Prado-Wohlwend (22)	2022	R	9	45.8 (20–72)	M 4, F 5	^177^Lu	7.4–8.4	3 (1-4)	29.78 (4–81)
Severi (23)	2021	P	12	52	M 20, F 26	^90^Y	1.1 or 1.85	5	76 (5-91)
			34			^177^Lu	3.7 or 5.5	5	73 (6-146)
Parghane (24)	2021	R	9	49 (33–61)	M 4, F 5	^177^Lu	5.55–7.4	4 (1-6)	(NA-40)
Roll (25)	2020	R	7	60 (14-84)	M 2, F 5	^177^Lu	NA	(3-5)	39
Jaiswal (26)	2020	R	15	32	M 7, F 8	^177^Lu	5.55-7.4	3 (1-6)	27 (11–62)
Zandee (27)	2019	R	30	47 (29–74)	M 20, F 10	^177^Lu	7.4	(2-4)	52.5 (7-155)
Vyakaranam (28)	2019	R	22	60 (24–80)	M 13, F 9	^177^Lu	7.4	4 (3-11)	32 (8–139)
Kolasinska-Ćwikła (29)	2019	P	13	42 (27–62)	M 8, F 5	^90^Y	3.4	(2-NA)	(0-140)
Yadav (30)	2019	R	24	35 (14–65)	M 19, F 6	^177^Lu	5.55-7.4	3 (2–8)	30 (15–96)
Demirci (31)	2018	R	12	NA	NA	^177^Lu	NA	(3-NA)	NA
Garske-Román (32)	2018	P	5	65 (25-71)	M 2, F 3	^177^Lu	7.4	(6-8)	NA
Kong (33)	2017	R	20	(21-77)	M 13, F 7	^177^Lu	NA	4	28 (5-74)
Mohammadali (34)	2017	P	5	NA	NA	^177^Lu	7.4	4	31 (1-168)
Pinato (35)	2016	R	5	29 (16-47)	M 4, F 1	^177^Lu	6.6-7.6	NA	NA
Nastos (36)	2016	R	11	41 (29-63)	M 6, F 5	^90^Y/^177^Lu	NA	(1-3)	NA
Puranik (37)	2015	P	9	(30-75)	M 4, F 5	^90^Y/^177^Lu	NA	2 (2-4)	(NA-6)
Imhof (38)	2011	P	39	NA	NA	^90^Y	NA	2 (1-10)	NA
Forrer (39)	2008	R	28	42 (16-69)	M 12, F 16	^90^Y/^177^Lu	NA	NA	49
Essen (40)	2006	R	12	39 (22-55)	M 6, F 6	^177^Lu	NA	NA	NA

R, Retrospective; P, Prospective; M, Male; F, Female; NA, not available.

**Table 2:** Quality assessment of the included studies based on the Newcastle-Ottawa Scale.

First author	Selection	Comparability	Outcome	Total score
Yadav (16)	3	1	2	6
Prado-Wohlwend (22)	3	1	3	7
Severi (23)	3	1	3	7
Parghane (24)	3	1	3	7
Roll (25)	3	1	3	7
Jaiswal (26)	3	1	3	7
Zandee (27)	3	1	3	7
Vyakaranam (28)	3	1	3	7
Kolasinska-Ćwikła (29)	3	1	2	6
Yadav (30)	3	1	3	7
Demirci (31)	3	1	2	6
Garske-Román (32)	3	1	2	6
Kong (33)	3	1	2	6
Mohammadali (34)	3	1	3	7
Pinato (35)	3	1	2	6
Nastos (36)	3	1	2	6
Puranik (37)	3	1	2	6
Imhof (38)	3	1	2	6
Forrer (39)	3	1	3	7
Essen (40)	3	1	2	6

### 3.3 Pooled analysis of efficacy

**Figure 2:**
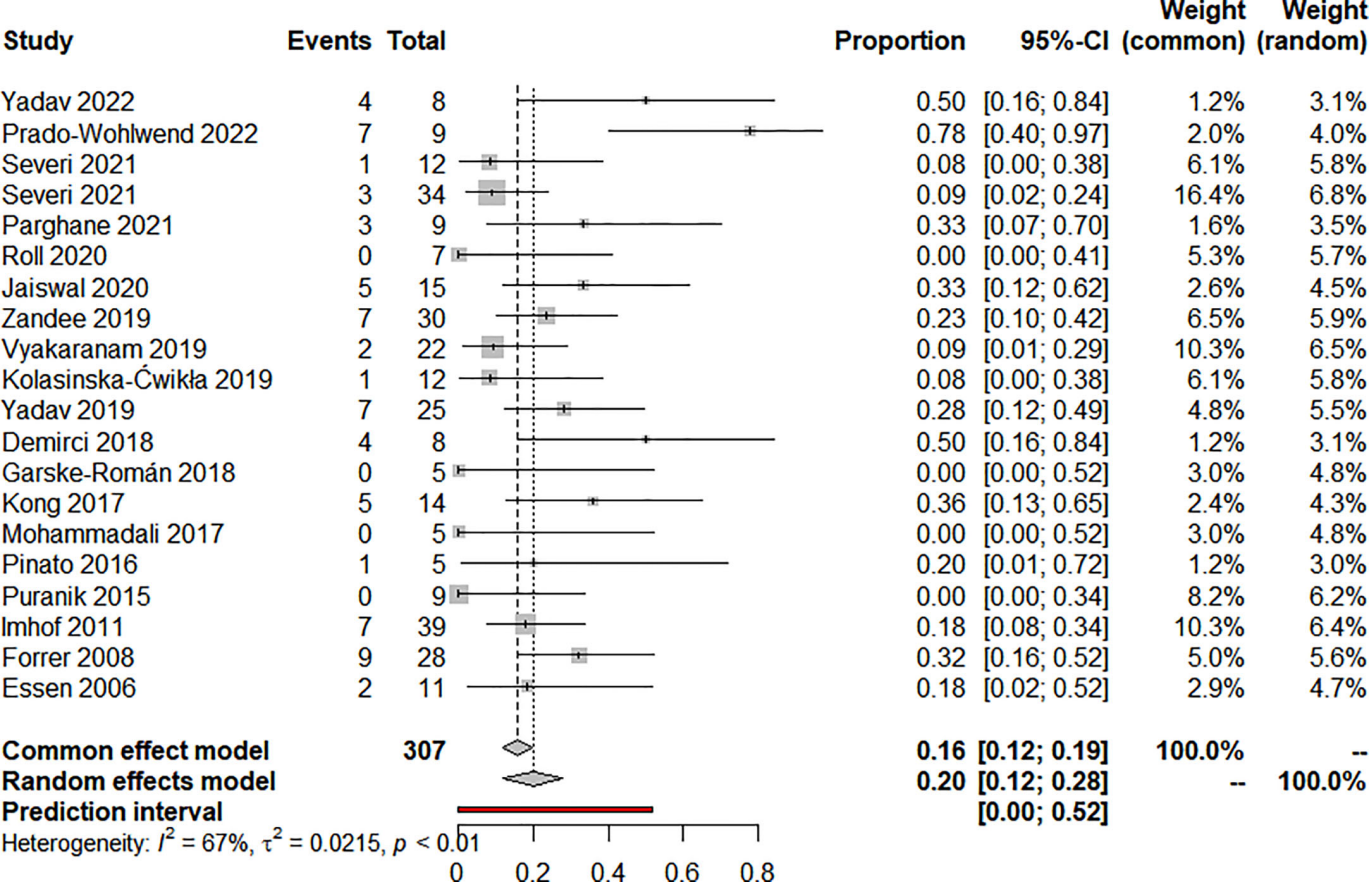
Forest plot for objective response rate after treatment.

**Figure 3:**
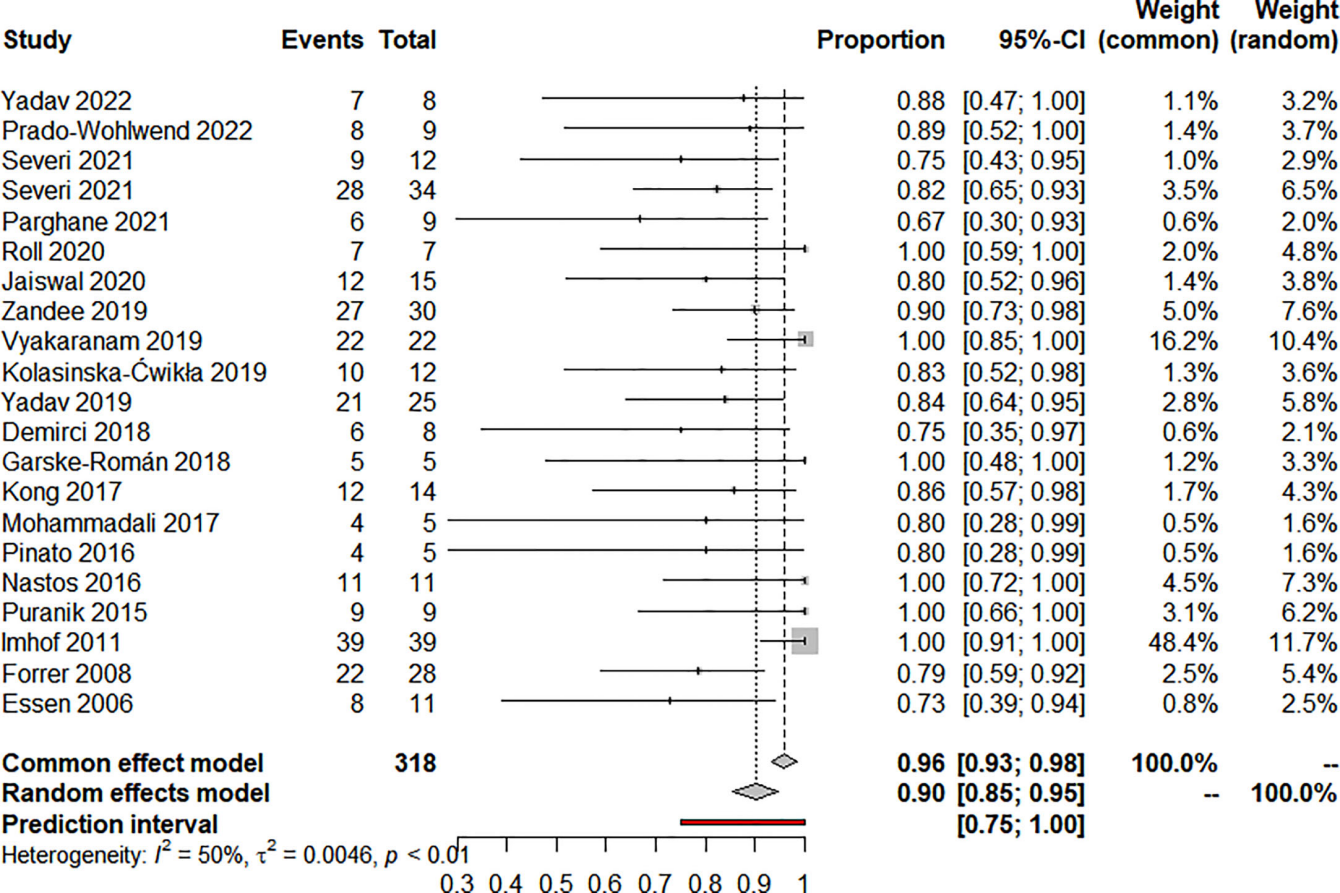
Forest plot for disease control rate after treatment.

Nineteen articles reported on ORR and 20 articles reported on DCR, eighteen used RECIST, and two used the SWOG criteria. The pooled analysis showed that the pooled ORR and DCR were 20.0% (95% CI: 12.0%-28.0%) (**Figure 2**) and 90.0% (95% CI: 85.0%-95.0%) (**Figure 3**), respectively. Clinical and biochemical responses were examined in 11 and 8 articles, respectively, with pooled proportions of 74.9% (95% CI: 56.3%-90.2%) and 69.5% (95%CI: 40.2%-92.9%). Thirteen articles reported median PFS, two of which did not reach median PFS at follow-up, and two articles fully reported the median PFS and 95% confidence interval, with a pooled estimate of 31.79 (95% CI: 21.25-42.33) months. Besides, 11 articles reported median OS, two of which did not reach median OS at follow-up, and four articles fully reported the median OS and 95% confidence interval, with a pooled estimate of 74.30 (95% CI: 0.75-147.84) months. All of the included studies’ efficacy results are shown in **Table 3**.

**Table 3:** Efficacy data of PRRT in the included studies.

First author	Criteria	ORR, n/N (%)	DCR, n/N (%)	Clinical response, n/N (%)	Biochemical response, n/N (%)	PFS, (Median) (Months)	OS, (Median) (Months)
Yadav (16)	RECIST	4/8 (50)	7/8 (87.5)	7/9 (77.8)	NA	NA	NA
Prado-Wohlwend (22)	RECIST	7/9 (77.8)	8/9 (88.9)	NA	NA	29 (Range 0–81)	NA
Severi (23)	SWOG	1/12 (8.3)	9/12 (75)	NA	NA	74.5 (95% CI: 8.4-NR)	92 (95% CI: 57.1-92.1)
		3/34 (8.8)	28/34 (82.4)	NA	NA	NA	143.5 (95% CI: 143.5-146.2)
Parghane (24)	RECIST	3/9 (33.3)	6/9 (66.7)	8/9 (88.9)	6/9 (66.7)	NR	NR
Roll (25)	RECIST	0/7 (0)	7/7 (100)	3/7 (42.9)	NA	NA	NA
Jaiswal (26)	RECIST	5/15 (33.3)	12/15 (80)	13/15 (86.7)	5/9 (55.6)	NR	NA
Zandee (27)	RECIST	7/30 (23.3)	27/30 (90)	6/7 (85.7)	3/6 (50)	NA	NA
Vyakaranam (28)	RECIST	2/22 (9.1)	22/22 (100)	8/10 (80)	12/15 (80)	21.6 (Range:6.7–138)	49.6 (Range:8.2–139)
Kolasinska-Ćwikła (29)	RECIST	1/12 (8.3)	10/12 (83.3)	11/13 (84.6)	4/4 (100)	35.0 (95% CI:24.4–93.1)	68.0 (95% CI:38.6–105.1)
Yadav (30)	RECIST	7/25 (28)	21/25 (84)	6/14 (42.9)	23/25 (92)	32	16 (95% CI: 14–24)
Demirci (31)	RECIST	4/8 (50)	6/8 (75)	NA	NA	31.405 (95%CI:20.331-42.478)	51.765 (95%CI:39.777-63.753)
Garske-Román (32)	RECIST	0/5 (0)	5/5 (100)	NA	NA	14 (Range 12-NR)	37 (Range 16-54)
Kong (33)	RECIST	5/14 (35.7)	12/14 (85.7)	15/17 (88.2)	12/14 (85.7)	39	NR
Mohammadali (34)	RECIST	0/5 (0)	4/5 (80)	NA	NA	NA	NA
Pinato (35)	RECIST	1/5 (20)	4/5 (80)	NA	NA	17 (Range 0-78)	53
Nastos (36)	RECIST	NA	11/11 (100)	NA	NA	38.5	60.8
Puranik (37)	RECIST	0/9 (0)	9/9 (100)	9/9 (100)	NA	NA	NA
Imhof (38)	RECIST	7/39 (17.9)	39/39 (100)	11/39 (28.2)	6/39 (15.4)	NA	82 (Range 56‐109)
Forrer (39)	RECIST	9/28 (32.1)	22/28 (78.6)	NA	NA	(Range 3-42)	NA
Essen (40)	SWOG	2/11 (18.2)	8/11 (72.7)	NA	NA	NA	NA

NR, not reached; NA, not available.

### 3.4 Pooled analysis of toxicity

A total of 17 articles reported data on toxicity. Analysis of 270 patients from 16 articles showed that 22.3% (95% CI: 12.5%-33.5%) of patients developed any grade of hematotoxicity and 4.3% (95% CI: 0.2%-11.4%) of patients developed grade 3-4 hematotoxicity. Analysis of 212 patients from 12 articles showed that nephrotoxicity of any grade occurred in 1.9% (95% CI: 0.0%-6.2%) of patients, and grade 3-4 in 4 out of 212 patients. The pooled estimate for treatment discontinuation was 9.0% (95% CI: 0.5%-23.3%), with most patients due to disease progression or upper renal dose limits. Three patients discontinued treatment due to recurrent thrombocytopenia, while one patient discontinued treatment due to nephrotoxicity after completing the third PRRT cycle (cumulative dose was 11.1GBq). Additionally, one patient had myelodysplastic syndrome (MDS) after receiving a cumulative dose of 44.4GBq and passed away 4.5 years later from complications related to MDS. Detailed data on toxicity are shown in **Table 4**.

**Table 4:** Toxicity profile of PRRT in the included studies.

First author	Criteria	Hematotoxicity, n/N (%)	Nephrotoxicity,n/N (%)	Treatment stopped, n/N (%)	Treatmentrelated deaths, n/N (%)
Yadav (16)	CTCAE	0	0	Disease progression 1/9 (11.1); Depression 1/9 (11.1)	0
Prado-Wohlwend (22)	CTCAE	G1-2 Anemia, Thrombocytopenia and Leucopenia 3/9 (44.44); G3-4 Leucopenia 1/9 (11.1)	NA	Disease progression 3/9 (33.3)	0
Severi (23)	CTCAE	G1-2 23/47 (48.9)	G1-2 3/47 (6.4);	nephrotoxicity 1/47 (2.13)	0
Parghane (24)	CTCAE	G1-2 Anemia 1/9 (11.1)	0	0	0
Roll (25)	CTCAE	G1-2 Anemia and Leucopenia 3/7 (42.9)	NA	NA	NA
Jaiswal (26)	CTCAE	G1-2 Anemia and Thrombocytopenia 3/15 (20)	0	0	0
Zandee (27)	CTCAE	G3-4 Anemia, Thrombocytopenia and Leucopenia 10/30 (33.3)	NA	thrombocytopenia 3/30 (10)	MDS 1/30 (3.3)
Vyakaranam (28)	CTCAE	G1-2 Anemia and Leucopenia 12/22 (54.5)	0	Renal dose reached the upper limit 17/22 (77.3); Disease progression 2/22 (9.1)	0
Kolasinska-Ćwikła (29)	CTCAE	G3 Anemia 2/13 (15.4)	G3 2/13 (15.4)	Patholodic spine fracture 1/13 (7.7)	0
Yadav (30)	CTCAE	G1-2 Lymphocytopenia 3/25 (12)	0	NA	0
Kong (33)	CTCAE	G3 Thrombocytopenia and Lymphocytopenia 4/19 (21.1)	G3-4 1/19 (5.3)	0	0
Mohammadali (34)	CTCAE	NA	NA	0	0
Pinato (35)	CTCAE	0	0	Pneumonitis 1/5 (20)	NA
Nastos (36)	CTCAE	G2 Anemia, Neutropenia and Thrombocytopenia 3/11 (27.3); G3-4 Thrombocytopenia, Neutropenia and Lymphocytopenia 4/11 (36.4)	G3-4 4/11 (36.4)	0	NA
Puranik (37)	CTCAE	0	0	NA	NA
Forrer (39)	CTCAE	G1 Anemia and Thrombocytopenia 2/28 (7.1)	0	0	NA
Essen (40)	CTCAE	G3-4 Thrombocytopenia and Anemia 2/12 (16.7)	NA	Persistent thrombocytopenia and anemia 2/12 (16.7)	NA

NA, not available.

### 3.5 Heterogeneity

There was clinical heterogeneity between included studies in terms of study design and PRRT treatment parameters. Statistical heterogeneity was significant in the combined effects of ORR (I^2 ^= 67%, P<0.01), DCR ((I^2 ^= 50%, P<0.01), clinical response rate (I^2 ^= 79.5%, P<0.01) and biochemical response rate (I^2 ^= 88.3%, P<0.01). Similarly, statistical heterogeneity was significant in the combined effects of median OS (I^2 ^= 99.8%, P<0.01), hematotoxicity at any level (I^2 ^= 71.5%, P<0.01) and treatment discontinuation (I^2 ^= 85.6%, P<0.01). median PFS (I^2 ^= 0.0%, P=0.824) and nephrotoxicity (I^2 ^= 37.2%, P=0.093) were not statistically significantly heterogeneous in the combined effects.

## 4 Discussion

Currently, PRRT on NETs is being tested in different countries, showing encouraging results. However, as most trials were small samples, single-institutional, and about mixed-type NETs, there are few systematic reviews of PRRT for PPGLs. This meta-analysis evaluated the efficacy and safety of PRRT in patients with advanced PPGLs from studies published to date. Most patients with advanced PPGLs were controlled after PRRT, and the overall DCR was 90.0%, with good clinical and biochemical response rates and prolonged survival. In the studies, most patients received other treatments (surgery, radiotherapy, chemotherapy, ^131^I-MIBG) before PRRT, and the results showed that PRRT still has some efficacy in patients whose disease progressed after these treatments. Interestingly, in the study of Yadav et al. (16), ^225^Ac-DOTATATE therapy was a salvage treatment option in patient refractory to ^177^Lu-PRRT. Their study provides a framework for a new promising aspect of treatment of advanced-stage PGLs. Moreover, the preliminary results of this pilot study provide a blueprint and encourage to conduct future prospective, two-armed randomized control studies on the head-to-head comparison between beta-particle-based ^177^Lu-DOTATATE and alpha-particle-based ^225^Ac-DOTATATE treatment in PGLs.

SSTR targeted imaging is the basis of PRRT by documenting adequate SSTR expression, which is the target of therapy. The Krenning score was used to grade the affinity of radiotracer in ^111^In-Pentetreotide imaging (40) and was later modified for SSTR PET. In principle, most patients with no or low affinity on SSTR PET should not be considered for PRRT. The expression of SSTR in lesions should not be used as an indication for PRRT, whether visually or based on standardized uptake value (SUV), and the clinical context must always be considered when selecting treatment, while taking advantage of the unique advantages of systemic evaluation of SSTR PET.

Large differences in survival in patients with pheochromocytoma and paraganglioma are related to several variables, including genetic status, size of the primary tumor, biochemical phenotype, and presence of metastatic disease at initial diagnosis (41, 42). Because current treatment of metastatic disease is rarely curative, systemic therapy is also an option for inoperable or metastatic disease that is progressive or has refractory symptoms. The most common chemotherapy regimen for systemic treatment is dacarbazine, cyclophosphamide and vinblastine, which may be chosen for rapidly developing diseases (11). Sunitinib is one example of a tyrosine kinase inhibitor that targets the vascular endothelial growth factor pathway (43). External radiation is typically effective for local management of diseases with localized symptoms (44). HSA ^131^I-MIBG and PRRT are options for less urgent treatments. Currently, the US FDA has only approved HSA ^131^I-MIBG for the treatment of pheochromocytoma and paraganglioma.

Regarding the adverse events (toxicity) of treatment, the most common adverse effect observed in all studies was hematotoxicity, which was mild in most patients. Grade 3-4 hematotoxicity in most patients was thought to be associated with prior chemotherapy (22, 29, 36), and PRRT was rarely discontinued. Besides, few patients developed nephrotoxicity after treatment. In addition, a hormonal crise caused by radionuclide therapy is a real problem with PPGLs, such as hypotension and myocardial ischemia in one patient with metastatic PCC one day after ^177^Lu-DOTATATE therapy (45). In the study of Makis et al. (46), two patients were also described with acute catecholamine crises within hours and 3 days after treatment, respectively. Of course, it is necessary to pay close attention to fluctuations in blood pressure control after each treatment. Pulmonary toxicity and severe pain associated with reactive tumor swelling have also been reported (35). Current studies showed that PRRT could be tolerated by most patients, but there was fewer data on long-term adverse events after treatment.

The current empirical treatment regimen for ^177^Lu-DOTATATE is 4 cycles of 7.4 GBq each, administered every 2 months, to avoid exceeding dose limits in critical organs. There is growing evidence to support patient-based individualized PRRT therapy, which can not only optimize the absorbed dose of tumor, but also limit radiation dose to critical organs. It has been suggested that dosimetry-based optimization methods should be added to the registry in addition to fixed treatment regimens to enable clinical dosimetry (47). Individualized PRRT therapy using renal dosimetry appears to be feasible and safe, and could result in an increased number of treatment cycles for most patients (48). Individualized PRRT based on renal absorbed dose results in an average 1.48-fold increase in cumulative maximum tumor absorbed dose over empirical PRRT, which may lead to increased response to treatment in clinical situations (49). In addition, somatostatin receptor PET imaging can predict tumor absorption dose (50). Pretreatment ^68^Ga-DOTATATE PET renal uptake can be used to predict ^177^lu-PRRT SPECT-derived mean renal absorbed dose with an average accuracy of within 18% (51). As the majority of patients treated with PRRT are those with advanced tumors, individualized therapy may prolong survival in relatively healthy conditions.

Our meta-analysis has some limitations as well. The included data had a high inherent bias risk since they lacked outcomes from randomized controlled trials. Clinical heterogeneity was still present despite the random-effects model’s ability to explain statistical heterogeneity. Besides, the majority of articles don’t use consistent standards for evaluating clinical and biochemical responses. Additionally, few studies estimate survival and offer information on long-term adverse effects. Nonetheless, our analysis results show good consistency compared with previous studies, indicating that PRRT has a good therapeutic effect in advanced PPGLs.

## 5 Conclusion

Peptide receptor radionuclide therapy has achieved a significant role in patients with advanced pheochromocytomas or paragangliomas. To further assess its efficacy and safety, prospective, multi-armed, multi-center randomized controlled studies are required.

## Data availability statement

The original contributions presented in the study are included in the article/supplementary material. Further inquiries can be directed to the corresponding author.

## Author contributions

DS and HY wrote this manuscript together. DS, HY and CQ collected relevant information. YC revised the manuscript finally and provided some critical suggestions. All authors listed have read and approved this article.
